# Altered Patterns of Reflex Excitability, Balance, and Locomotion Following Spinal Cord Injury and Locomotor Training

**DOI:** 10.3389/fphys.2012.00258

**Published:** 2012-07-18

**Authors:** Prodip K. Bose, Jiamei Hou, Ronald Parmer, Paul J. Reier, Floyd J. Thompson

**Affiliations:** ^1^Brain Rehabilitation Research Center, North Florida/South Georgia VA Medical CenterGainesville, FL, USA; ^2^Department of Physiological Sciences, University of FloridaGainesville, FL, USA; ^3^Department of Neurology, University of FloridaGainesville, FL, USA; ^4^Department of Neuroscience at the University of FloridaGainesville, FL, USA

**Keywords:** spasticity, ankle torque, EMG, locomotor training, spinal cord injury, rat

## Abstract

Spasticity is an important problem that complicates daily living in many individuals with spinal cord injury (SCI). While previous studies in human and animals revealed significant improvements in locomotor ability with treadmill locomotor training, it is not known to what extent locomotor training influences spasticity. In addition, it would be of considerable practical interest to know how the more ergonomically feasible cycle training compares with treadmill training as therapy to manage SCI-induced spasticity and to improve locomotor function. Thus the main objective of our present studies was to evaluate the influence of different types of locomotor training on measures of limb spasticity, gait, and reflex components that contribute to locomotion. For these studies, 30 animals received midthoracic SCI using the standard Multicenter Animal Spinal cord Injury Studies (MASCIS) protocol (10 g 2.5 cm weight drop). They were divided randomly into three equal groups: control (contused untrained), contused treadmill trained, and contused cycle trained. Treadmill and cycle training were started on post-injury day 8. Velocity-dependent ankle torque was tested across a wide range of velocities (612–49°/s) to permit quantitation of tonic (low velocity) and dynamic (high velocity) contributions to lower limb spasticity. By post-injury weeks 4 and 6, the untrained group revealed significant velocity-dependent ankle extensor spasticity, compared to pre-surgical control values. At these post-injury time points, spasticity was not observed in either of the two training groups. Instead, a significantly milder form of velocity-dependent spasticity was detected at postcontusion weeks 8–12 in both treadmill and bicycle training groups at the four fastest ankle rotation velocities (350–612°/s). Locomotor training using treadmill or bicycle also produced significant increase in the rate of recovery of limb placement measures (limb axis, base of support, and open field locomotor ability) and reflex rate-depression, a quantitative assessment of neurophysiological processes that regulate segmental reflex excitability, compared with those of untrained injured controls. Light microscopic qualitative studies of spared tissue revealed better preservation of myelin, axons, and collagen morphology in both locomotor trained animals. Both locomotor trained groups revealed decreased lesion volume (rostro-caudal extension) and more spared tissue at the lesion site. These improvements were accompanied by marked upregulation of BDNF, GABA/GABA_b_, and monoamines (e.g., norepinephrine and serotonin) which might account for these improved functions. These data are the first to indicate that the therapeutic efficacy of ergonomically practical cycle training is equal to that of the more labor-intensive treadmill training in reducing spasticity and improving locomotion following SCI in an animal model.

## Introduction

Spinal cord injury (SCI) produces a number of complicated challenges to the recovery of locomotor function, particularly, re-training the residual nervous system to overcome obstacles posed by the loss of connectivity diminished by injury or enhanced by non-adaptive plasticity. The fundamental locomotor disabilities span a wide range and include spasticity and balance instability. Spasticity is a disability that occurs in a high proportion of SCI patients. While often complicated in form and content, the clinical hallmark of spasticity is a significant exaggeration of the velocity-dependent lengthening resistance of the affected muscles (Lance, 1980; Young, 1989a,b). Inappropriate resistance to movement, painful spasms, and movement interference associated with spasticity complicate the quality of life and contribute barriers to locomotor recovery (Katz and Rymer, 1989). In addition to spasticity, there are additional factors in the post-SCI setting that may influence limb use; postural instability associated with changes in descending modulation of balance may produce compensatory changes in base of support and limb axis. Profound atrophy of locomotor skeletal muscle is a potentially serious complication that may further challenge locomotor recovery as well as contribute to metabolic changes following SCI (Houle, 1999; Hutchinson et al., 2001; Gregory et al., 2003; Haddad et al., 2003; Stevens et al., 2006; Liu et al., 2008, 2010; Shah et al., 2008).

Rehabilitation strategies utilizing locomotor activity to direct constructive plasticity of spinal cord locomotor circuits have revealed encouraging breakthroughs in the potential for locomotor recovery using treadmill and, less frequently, stationary bicycle, training programs. Recent evidence indicates that individuals with complete and incomplete SCIs improve their ability to step on a treadmill, to cycle or walk overground following specific locomotor training (Visintin and Barbeau, 1989; Wernig and Muller, 1992; Dietz et al., 1994; Wernig et al., 1995; Harkema et al., 1997; Behrman and Harkema, 2000; and also see reviews, Barbeau et al., 1999; Basso, 2000; Wolpaw and Tennissen, 2001; Dietz and Harkema, 2004). Such locomotor training uses principles derived from animal and human studies showing that stepping can be generated by virtue of the neuromuscular system’s responsiveness to phasic, peripheral sensory information associated with locomotion (Lovely et al., 1986, 1990; Barbeau and Rossignol, 1987; Edgerton et al., 1997, 2004; Harkema et al., 1997; de Leon et al., 1998; Behrman and Harkema, 2000). This experimental regimen may promote the recovery of walking by optimizing the activity-dependent neuroplasticity of the nervous system produced by task-appropriate locomotor training (Edgerton et al., 1992; Muir and Steeves, 1997; Gomez-Pinilla et al., 2002). Neuronal circuits, stimulated by the proper activation of peripheral afferents via training, may reorganize by strengthening existing and previously inactive descending connections and local neural circuits (Edgerton et al., 1992; Dietz et al., 1997; Muir and Steeves, 1997; Barbeau et al., 1998; Basso et al., 2002). However, it is not known to what extent locomotor training influences lower limb spasticity and limb use parameters, or if there are significant differences in outcomes relative to the type of locomotor training used. Most of the rehabilitation studies (both clinical and laboratory) are largely accommodated by treadmill training programs. While treadmill training has been demonstrated to be effective, there are personnel, equipment, and space considerations associated with its use. By comparison, the stationary bicycle is spatially compact, economical to acquire, and can be safely accessed with minimal assistance. Therefore, it would be of importance to understand how bicycle locomotor training compares with treadmill training since there are several practical factors that make use of the bicycle training more accessible to SCI-individuals, particularly even in the home setting.

Studies in our laboratory have demonstrated the appearance of significant velocity-dependent lower limb spasticity, changes in limb placement during gait, and significant changes in excitability of stretch reflex pathways of lower limb muscles (Thompson et al., 1992, 1998; Bose et al., 2002). Collectively these changes represent robust and comprehensive changes consistent with a clinical definition of spasticity. The purpose of the present studies was to utilize these biomechanical, behavioral, and neurophysiological measures in this model of SCI to (1) provide preclinical data on quantitative assessment of the influence of locomotor training on lower limb spasticity, (2) to correlate these changes with neurophysiological processes that regulate reflex excitability, and (3) to compare potential benefits of treadmill vs. bicycle locomotor training.

## Materials and Methods

### Animal subjects

Thirty Sprague Dawley specific pathogen free (SPF) rats (12 weeks old, weighing 220–260 g at the start of this study; Charles Rivers Laboratory, USA) were used in this project. All procedures were performed in accordance with the U.S. Government Principle for the Utilization and Care of Vertebrate Animals and were approved by the Institutional Animal Care & Use Committee at the North Florida/South Georgia Veterans Health System and the University of Florida.

### Surgical procedure – contusion injuries

The contusion injuries were produced using a Multicenter Animal Spinal cord Injury Studies (MASCIS) impactor and protocol. Briefly, the MASCIS impactor, 10 g weight, was dropped from a 2.5 cm height onto the T_8_ segment of the spinal cord exposed by laminectomy under sterile conditions. Each animal received Ampicillin (s.q.) twice each day starting at the day of surgery for a total of 5 days. The procedure was performed under ketamine (100 mg/kg)-xylazine (6.7 mg/kg) anesthesia (details in Reier et al., 1992; Thompson et al., 1992, 1993; Bose et al., 2002). The animals were kept under vigilant post-operative (po) care which included daily examination for signs of distress, weight loss, dehydration, and bowel and bladder dysfunction. Manual expression of bladders was performed 2–3 times daily as required, and the animals were monitored for the possibility of urinary tract infection. Animals were housed in pairs (except for a brief po recovery period). At po day 7, velocity-dependent ankle torque (Bose et al., 2002) and open field locomotion were assessed by the Basso, Beattie, Bresnahan (BBB) scoring scale (Basso et al., 1995) to obtain measures of spasticity and injury severity, respectively. If any animal does not fall within certain preset scores (ankle torque at 612°/s angular rotation, 160–220 kdyne, and BBB scores >5 at po day 7), it was considered too mildly injured and eliminated from the study to reduce the variability. Out of a total of 42 animals, 30 animals were qualified for the preset criteria. The animals (*n* = 30) were then randomly divided into three equal groups (*n* = 10 each). Two groups were assigned for treadmill and cycling locomotor trainings, and, the third group did not receive training (contused control for both groups), however, was checked routinely.

### Locomotor training

A three-runway treadmill (Columbus Instrument, OH, USA) and two custom made bicycles were used in this study for locomotor training.

### Rat bicycle

**Figure 1:**
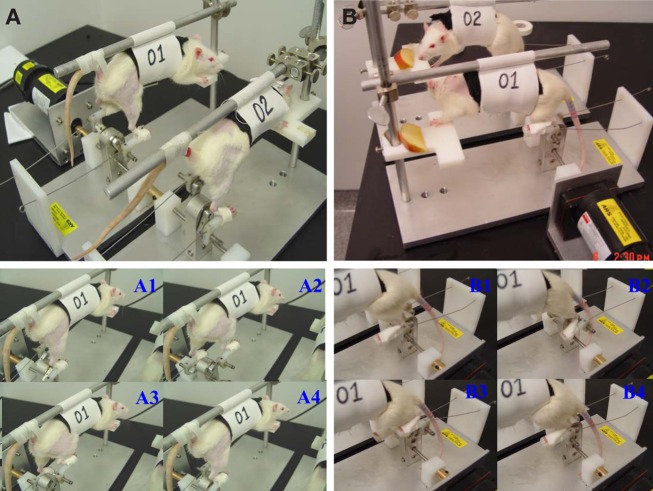
**A custom made motorized rat bicycle (A,B), and a series of sequential video images of spinal cord-contused rats cycling on this apparatus**. Note, right hindlimb was shaved for EMG recording. During the first week of training, the tail was taped **(A, A1–A4)** to provide maximum body support. However, the body support was gradually reduced to maximize the loading during training after first week **(B, B1–B4)**.

A rodent motorized rat bicycle was designed and custom built to promote locomotion following SCI (University of Florida patent pending; Application number: 61698752). The bicycle is composed of a direct drive gear box, adjustable foot pedals, and a support harness. Two pedal guide-wires are placed to maintain proper pedal orientation. The drive shaft ends are keyed to allow multiple bikes to operate in series on a single drive motor. The assembly is mounted on a thick aluminum base plate for added stability and strength (see Figure 1).

### Exercise protocol

The animals were trained over the course of 3 months. The training schedule was performed 5 days a week using two 20 min trials/day, starting from po day 8 in both training paradigms. On the first day of training, the rats were given 5 min to explore the treadmill and then encouraged to walk on the moving treadmill (11 meter per minute, mpm, Kunkel-Bagden et al., 1993) for a series of 4, 5-min bouts of walking. The rats were given a minimum of 5 min rest between bouts. On the second day of training the rats walked for two bouts of 10 min each, twice a day, and then day 3–90, the rats were trained to walk for 20 min without a rest with a at least 2 h interval between trails. First 7 days bodyweight was supported as needed using support pole and harness attached with the support pole as seen in Figure 1. This allowed us to suspend the rat over the treadmill and provided similar bodyweight supported training like bicycle training. Moreover, this design of bodyweight support allowed us to assist limb locomotion with hands to promote normal walking during treadmill locomotion. The body support sling was positioned at a height such a way so that this could provide the desire body weight support/load during both types of exercise. After 7 days, the bodyweight was supported as needed. The bicycle exercise regimen involves suspending the rats on the rat harness (Figure 1) with the hindlimbs hanging down and hind feet strap onto the pedals with cotton tapes. The exercise consists of a pedaling motion, which fixed one limb while extending the other without overstretching the limbs. The cycling speed was 31 rotations/minutes (around 11 mpm distance wise). The first 2 days, the bicycle training period and protocol were same those of treadmill training. During the first week of training, the rat tail was attached with the aluminum support boom by surgical tape to maintain the trunk stability during exercise. However, following second week of training, gradually the load was increased by positioning the body harness toward the chest, so that the hind portion of the body falls over the pedal.

### Ankle torque and EMGs measurement

The lengthening resistance of the triceps surae muscles was measured indirectly by quantifying ankle torque and EMGs during 12° dorsiflexion ankle rotation (see detail in Thompson et al., 1996; Bose et al., 2002; Wang et al., 2002). This measurement is a standard procedure in our laboratory and detail is published, Bose et al. (2002) and Thompson et al. (1996). Prior to data acquisition, the animals was given a brief pre-recording period to adjust to the recording procedure by providing them with 12° ankle rotations produced at 3 s intervals at eight different velocities (49, 136, 204, 272, 350, 408, 490, and 612°/s). Rats were immobilized in a custom designed trunk restraint, without trauma or apparent agitation. All recordings were performed in awake animals. The proximal portion of the hind limbs to the midshank, were secured in a form-fitted cast that immobilized the limb while permitting normal range of ankle rotation (60–160°). The animals typically adjusted to the restraint device without detectable discomfort and were provided fruit to sniff or chew as a distraction. The neural activity of the triceps surae muscle was measured using transcutaneous EMG electrodes. The electrode was inserted in a skin fold over the distal soleus muscle just proximal to the aponeurotic convergence of the medial and lateral gastrocnemii into the tendonocalcaneousus. The reference electrode was placed in a skin fold over the greater trochanter. A xylocaine 2% jelly (Lidocaine HCl, Astra USA Inc.) was applied over the electrode insertion points to minimize pain during recording. The data recording session begins when the animal is relaxed and the protocol requires approximately 45 min. At each test velocity, five consecutive sets of waveforms, 10 waveforms per set (a total 50 waveforms/velocity), was recorded, signal averaged, and saved for subsequent analysis. A complete protocol for each animal was recorded during each of two separate recording sessions performed on separate days. Therefore, the data set for each animal for each test velocity was the signal average of 100 trials (50 per session × 2 sessions). The data were signal averaged upon acquired using a digital signal acquisition system and LabView graphic programming (Version 5.0, National Instrument).

### Rate-depression

Measurement of rate-depression is a well-established model in our laboratory (Thompson et al., 1992, 1993, 1998, 2001a). Rate-depression was assessed using a non-invasive procedure. The animals were anesthetized by i.p. injection of ketamine 100 mg/kg and immobilized in a prone position on the recording table using surgical tape. Ketamine was selected due to its minimal depression of monosynaptic reflex and because it does not alter the time course of presynaptic inhibition (Lodge and Anis, 1984). Core body heat was maintained via heat lamp. The hair overlying the distal tibial nerve at the ankle was removed using a cosmetic hair removing gel. A bipolar stimulating electrode with 1 mm silver ball was applied to the ankle surface and just enough electrode gel to coat the tip of the electrode applied to the skin. A monopolar surface EMG recording electrode was applied to the plantar skin overlying the lateral plantar (digital interosseus) muscles. The reference was applied to the skin surface of the fifth digit. A ground electrode was applied to the skin surface intermediate between the stimulating and the recording electrode to minimize shock artifact. The distal tibial nerve was stimulated using 200μsec current pulses, according to a preset protocol to determine H-reflex threshold and H-max, M-wave threshold and M-max. An H-recruitment curve was then made to locate the minimum intensity for the maximal reflex amplitude. The frequency protocol was performed at this intensity and was adjusted slightly during the frequency series to maintain a constant M-wave amplitude (an assurance of a constant effective stimulus delivery to the distal tibial nerve). The frequency series was included 0.3 Hz as control with 7 test frequencies: 0.5, 1, 2, 3, 4, 5, and 10 Hz. The data set for each frequency was 32 consecutive waveforms that were signal averaged upon acquired using a digital signal acquisition system and LabView graphic programming (Version 5.0, National Instrument). Rate-depression at each test frequency was quantified by comparison of reflex amplitude and area to the 0.3 Hz control.

### Footprints

Graph paper was placed on the treadmill and the rats’ hind limbs were inked. The rats were then placed on the treadmill (20 × 40 cm) surface at the practiced speed, 11 mpm. Axial angle of rotation, and base of support were analyzed from these footprints. Angle of rotation is the angle measurement found by drawing a line through the center of the third toe and the center of the heel of two consecutive paw prints. Base of support is the distance between two consecutive prints. Thus, hind limb gait abnormalities were measured from footprints obtained at preinjury, and 1–3 months flowing training using both trained and untrained contused animals.

### Open field locomotor recovery

Basso, Beattie, and Bresnahan open field locomotor scale was applied to score the early, intermediate, and late phases of recovery following locomotor training. The 21-point scale is based on the observation that after spinal cord contusion rats progressed through three general phases of recovery. The early phase is characterized by little or no hindlimb joint movement (scores 0–7). The intermediate phase includes bouts of uncoordinated stepping (scores 8–13), whereas the late phase involves fine details of locomotion such as dragging of the toes and tail, trunk instability, and rotation of the paws (scores 14–21). The animal was placed in a test apparatus, observed for 4-min, and scored in real time by two-blinded observers (Basso et al., 1995). All open field locomotor testing was video-taped for further analysis and review.

### Histological evaluation of lesion

The contusion lesions were studied histologically to assess the severity and nature of the injury using 4% buffered paraformaldehyde fixed tissues. The portions of the SC that included the contusion epicenter, and segments extending 4 mm rostral and caudal to the injuries were dissected and saved, embedded in paraffin, and sectioned on a Microtome (10 μm thickness; *n* = 6 in each group; control, treadmill, and cycle trained). The sections were stained using conventional Luxol fast blue and cresyl violet staining techniques. To quantify injury lesion length, the number of sections containing the lesions was counted from rostral to caudal on serial sections. The total number of injured sections was multiplied by the individual section thickness of 10 μm, in order to obtain total injury lesion length. Further quantification involved volumetric measurement of the injury lesions. In order to obtain volume measurements, the lesion area was measured in every tenth SC section (Noble and Wrathall, 1985). After obtaining lesion area, a previously published mathematical formula (Rosen and Harry, 1990) was applied to calculate the volume. Lesion areas equally placed at 100 μm was used in the Cavalier’s estimator of morphometric volume (Rosen and Harry, 1990): *V*_C_ = *d*[Σ(*y*_i_)] − (*t*)*y*_max_ where, “*V*_C_” is the Cavalieri’s estimator of volume; “*d*” is the distance between the sections being measured (100 μm); *y*_i_ is the cross-sectional area of the *i*-th section through the morphometric region; “*y*_max_” is the maximum area in the series, and “*t*” is section thickness (10 μm). The amount of spared white matter, both dorsal and ventral quadrants of the cavity, was also measured on the serial sections. The proportion of the residual ventral white matter was expressed in relation to that observed in intact normal animal’s tissue (100% spared ventral white matter). Light microscopic qualitative studies were also conducted to assess the morphology of the spared tissue. This goal was achieved by examining the extent of myelination, characteristics of remaining axons, and the degree of collagen infiltration. Normal-appearing gray matter was distinguished from damaged tissue by the presence of healthy neurons and normal cellular density (without the presence of numerous nuclei which is indicative of immune cell infiltration). Normal-appearing white matter was defined as being non-fragmented, darkly bluestained (not pale blue), and without immune cell infiltration (Pearse et al., 2004).

### Immunohistochemistry

Spinal cord segments caudal to the injuries, and lumbar spinal cord, L_3_-L_6_) were dissected and removed after perfusion and kept in the same fresh fixative mixture for 1 h and were cryoprotected for at least 2 days in 30% sucrose in phosphate buffer (PB). The specimens were cut serially (cross section) by cryostat (40 μm thickness) and processed by Avidine-Biotine Complex (ABC) and fluorescent immunohistochemistry (IHC). The immunoreactivity of GAD_67_, GABA_b_, and dopamine-beta-hydroxylase (DBH, for NE descending projections) and BDNF were identified in SC. The cryostat cut sections were incubated with primary antibodies generated against GAD_67_ (mouse mAb; 1:1,000, National Hybridoma Laboratory, St Louis, USA), GABA_b_ (guinea pig mAb, 1:4,000; Chemicon International), DBH (mouse mAb, 1:7000; Chemicon International), a synthetic enzyme found in the neurotransmitter vesicles of noradrenergic fibers, and BDNF (rabbit Ab, Chemicon International) for 24–48 h at 4°C. The sections were then washed in PBS and incubated for 1.5 h in alexa fluor-conjugated appropriate anti-mouse, anti-guinea pig, or anti-rabbit IgG (1:1,000, Molecular Probes). For ABC technique, anti-guinea pig (1:200; Chemicon), anti-mouse, and anti-rabbit (1:200; mouse and rabbit Elite kits, Vector Lab) secondary antibodies were used to bind with appropriate primary antibodies. Co-localization of GAD_67_, BDNF, and DBH labeled cells were also performed by double labeling with the appropriate antibodies using the same procedure. Sections were washed again and mounted for microscopic analyses.

### Statistical analyses

Analysis of variance (ANOVA) was used to detect differences in ankle torques and EMGs values obtained at each velocity from precontused, contused, treadmill, and cycle exercised animals. Ankle torques and corresponding EMG values were obtained at pre-injury time point from each group were also tested by ANOVA. In addition, a repeated measures ANOVA (RM ANOVA) was used to test the within group differences in ankle torque or EMGs across po weeks. Data from H-reflex, footprints, BBB, and histological experiments were analyzed by using ANOVA to assess treatment effects from contused and time-matched normal control groups. The level of significant difference was set for all analysis was *p* ≤ 0.05. Significant differences are marked with asterisks (*) or ^^^ according to their respective *p*-values: *,compared with pre-injury values; ^^^,compared with contused controls. Values are expressed as the mean value ± standard error of the mean (SEM) in all graphs.

## Results

### Velocity-dependent ankle torque and associated EMGs

**Figure 2:**
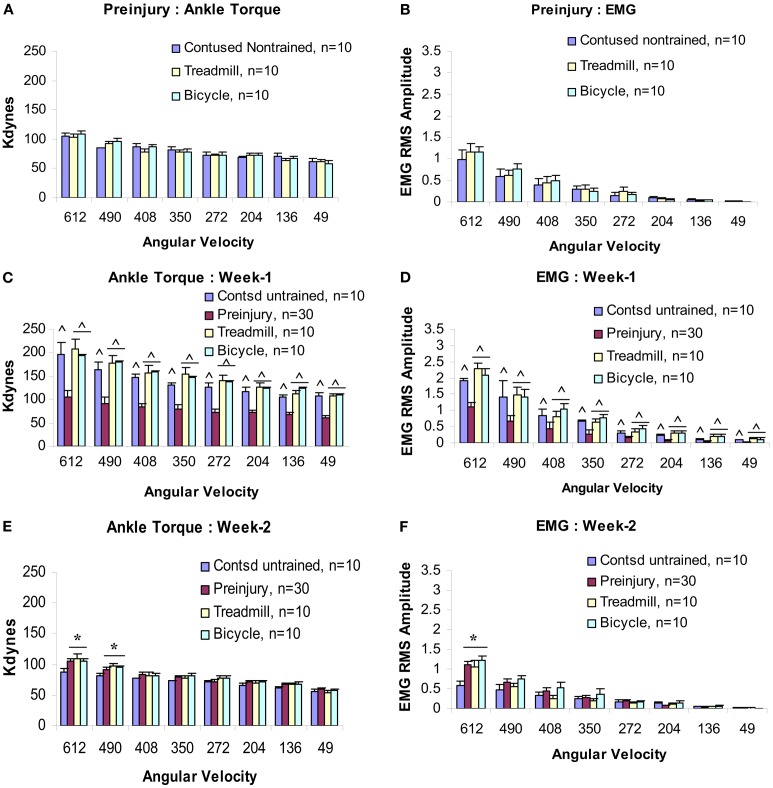
**Velocity-dependent ankle torque (A,C,E) and time-locked EMG-RMS magnitude (B,D,F) of precontusion and postcontusion weeks 1 and 2**. Note, both ankle torque and EMG-RMS values of postcontusion week 1 in all eight velocities (612–49°/s) were significantly greater compared with those of pre-contused values **(C,D)**. Interestingly, only 1 week of exercise (training started at p.o. day 8) prevents hypotonia **(E,F**; see text for detail). *p* < 0.05; *compared with controls; ^^^compared with preinjury.

Baseline measures of velocity-dependent ankle torques and extensor EMGs were obtained from all animals before injury (Figures 2A,B), at post-injury weeks 1 and 2, and then at alternate weeks up to po week 12. When tested at 1 week following injury, all three groups revealed significantly increased magnitudes of ankle torque during rotation at each of the eight ankle rotation velocities compared to the control values recorded before injury. There was no difference between these three groups, Figure 2C (ANOVA). The EMG-RMS magnitudes time-locked with increased ankle torques that were recorded at each of the test velocities were also significantly greater compared with those recorded before injury (Figure 2D).

At post-injury week 2, a pattern of significant hyporeflexia was observed in the untrained contused group, Figures 2E,F. However, ankle torque and triceps surae EMG magnitudes recorded from the two trained groups at the end of postcontusion week 2 did not demonstrate this pattern of hyporeflexia, but decreased from the week 1 values and were similar to data observed in precontusion animals (Figures 2E,F). Moreover, there was no difference between the data recorded from these two training groups at this post-injury time point.

**Figure 3:**
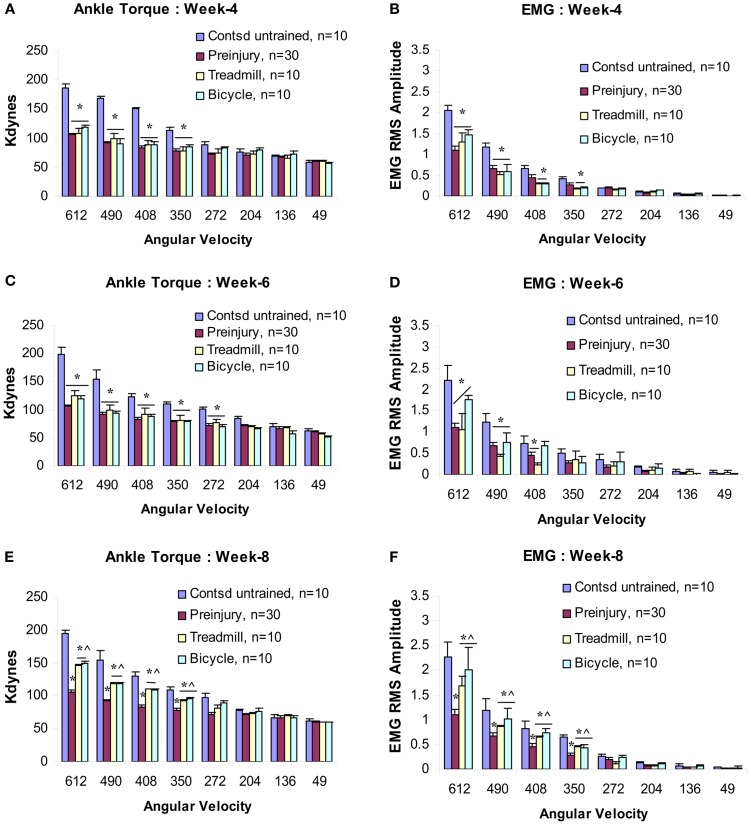
**Velocity-dependent ankle torque (A,C,E) and time-locked EMG-RMS magnitude (B,D,F) of postcontusion weeks 4–8**. Note, both type of trainings prevent development of spasticity up to postcontusion week 6 **(A–D)**, however, a milder form of spasticity (42% less than control) has been detected at postcontusion week 8 compared with untrained contused controls **(E,F)**. *p* < 0.05; *compared with controls; ^^^compared with preinjury.

By week 4 post-injury, a significant velocity-dependent ankle extensor spasticity re-appeared in the untrained contused group Figures 3A,B. However, this second appearance of spasticity occurred only during the faster ankle (dynamic) rotations and was no longer observed during the low velocity (tonic) rotations. Tests at all later time points revealed that this significant dynamic velocity-dependent increase in ankle torque was enduring. Surprisingly, at this post-injury time point, this re-emergent spasticity was not observed in either of the two training groups (Figures 3A,B). At this point, ankle torque and EMG magnitudes did not increase significantly at the four fastest ankle rotation velocities (350–612°/s) compared with the untrained contused animals (Figures 3A,B). Moreover, at the end of po week 6 (following 5 weeks of training), we did not observe increased ankle torque and EMG magnitudes at the four fastest ankle rotation velocities, as were clearly evident in the untrained contused animals (Figures 3C,D).

**Figure 4:**
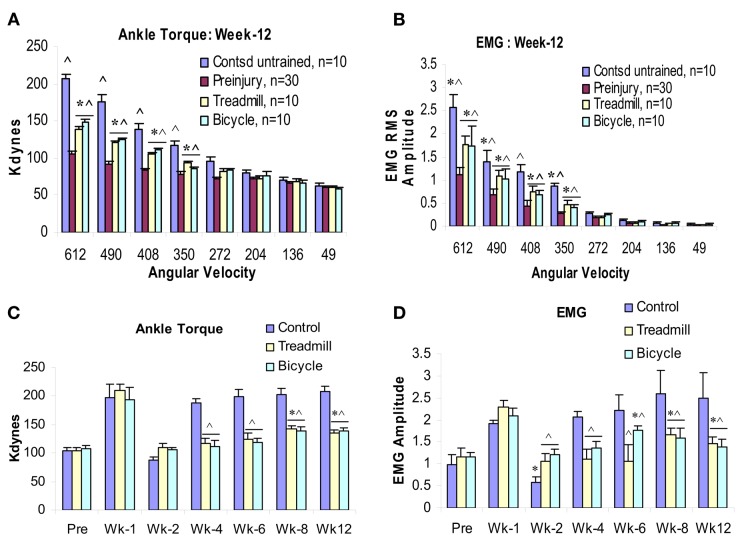
**Effects of 3 months locomotor training (treadmill and bicycle) on ankle torque (A), extensor muscle EMGs (B) in animals with midthoracic contusion injury**. Three-month locomotor training showed significant reduction of spasticity (ankle torque and EMGs) in both training groups (49% reduction). The time course of velocity-dependent ankle torque **(C)** and time-locked EMG-RMS magnitude **(D)** over 12 weeks. Both treadmill and bicycle training prevented the initial hyporeflexic state (at week 2), prolonged the transition to develop a permanent hyperreflexic state (weeks 4–6) and attenuated the level of spasticity (weeks 8–12).

At postcontusion weeks 8–12, an increase in the velocity-dependent ankle torque was observed in both treadmill and bicycle training groups. This appeared at the four fastest ankle rotation velocities (350–612°/s), and was of lower magnitude compared with values recorded in the untrained contused control group (Figures 3E,F and 4A–D). The mean torque and EMG values recorded at these rotation velocities from these trained groups were also increased and were intermediate in magnitude compared with corresponding values recorded from pre-injured normal and untrained contused groups. No significant increases in ankle torque or EMG magnitude was observed during ankle rotations at the slowest four velocities at postcontusion week 4–12 (Figures 3 and 4). It is important to note here also that there was no significant difference between the treadmill and bicycle group in ankle torques or EMGs recorded during post-injury week 4–12.

### Rate-depression of tibial/planter H-reflexes

**Figure 5:**
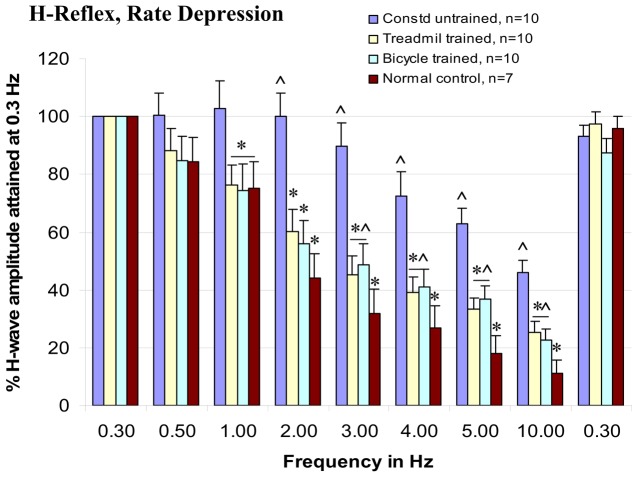
Both types of locomotor training enhance rate-depression of H-reflex at 1–10 Hz test frequencies, ANOVA, *p < 0.05, compared with contused untrained controls, and ^^^*p* < 0.05, compared with normal intact animals.

Rate-depression of the tibial/plantar H-reflexes was tested before injury and at 3 months post-injury in the trained and untrained injury groups. Compared with pre-injury controls, rate-depression was significantly reduced in the untrained group at each of the test frequencies from 1 to 10 Hz. By contrast, the rate-depression observed in the trained animals was intermediate in amplitude between the pre-injury controls and the untrained animals at 1–10 Hz, see Figure 4. Further, note that the rate-depression produced in the trained animals in response to test frequencies of 1–2 Hz, was similar to that recorded in the normal controls (Figure 5). Similar magnitudes of rate-depression were observed in bicycle and treadmill-trained animals.

### Gait and open field locomotion

#### Footprint analyses

**Figure 6:**
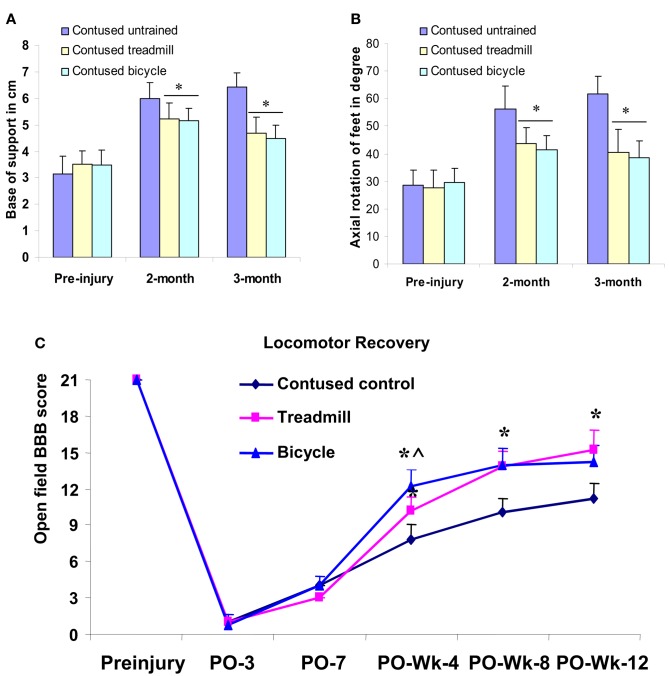
**Hind limb gait abnormalities and locomotor recovery measured from footprints and open field locomotor scores (BBB) and plotted as groups**. Locomotor training significantly reduced the deviation of axial hind limb rotation **(B)** and base of support **(A)** in both treadmill and bicycle training groups compared with untrained contusion group at second and third month (*p < 0.05 compared with contused untrained group). Both types of training significantly improved locomotor recovery compared with untrained controls **(C)**. The bicycle training group demonstrated the greatest recovery at postop 4 weeks, which is significantly different even compared with treadmill group. However, at postop 8 weeks, both training groups showed similar recovery. *p < 0.05 compared with untrained contused group; ^^^compared with treadmill group (ANOVA).

Footprint analyses were performed before injury, at 2 and 3 months post-injury. Before injury, limb axis and base of support were measured to be 30.25 ± 5.9 and 3.29 ± 0.51, respectively. By comparison, these measures were 55.78 ± 6.58 and 6.03 ± 0.42 in the untrained animals at 2 months post-injury. Similar values, 57.62 ± 6.84 and 6.52 ± 0.52, respectively, were measured at 3 months. Compared with the pre-injury control values, these measures revealed that limb axis and base of support were significantly increased in the untrained contusion-injured animals. In the 2–3 months locomotor training groups, limb axis and base of support were observed to be 41.37 ± 4.85 (at 2 months), 40.35 ± 5.87 (at 3 months) and 5.04 ± 0.32 (at 2 months), 4.52 ± 0.48 (at 3 months), respectively. These measures were significantly less altered than those observed in the untrained animals (Figures 6A,B). However, no significant difference in hind limb rotation or base of support was observed between the two training groups (ANOVA) at 2 or 3 months following training (Figures 6A,B).

### Open field locomotor recovery

Open field locomotor behavior was scaled (BBB) in both trained and untrained animals before injury and at post-injury 4, 8, and 12 weeks to evaluate recovery during the early, intermediate, and late phases of recovery.

At week 4, untrained contused animals displayed extensive movement of all three joints of the hindlimb, however, these animals could not support their body weight (mean score, 7.8 ± 1.2). In contrast, treadmill-trained animals showed occasional to frequent weight supported plantar steps, however, no forelimb-hindlimb (FL-HL) coordination (mean score, 10.2 ± 1.07) was observed. Interestingly, bicycle-trained animals displayed frequent to consistent weight supported plantar steps and occasional to frequent FL-HL coordination (mean score, 12.2 ± 1.4). This BBB score in the bicycle trained group was significantly greater (*p* < 0.05, ANOVA) than observed in either the untrained and the treadmill-trained groups (Figure 6C).

At postcontusion week 8, untrained control animals showed occasional to frequent weight supported plantar steps without FL-HL coordination (mean score 10.0 ± 1.24), whereas, both treadmill and bicycle-trained animals showed consistent weight supported plantar steps and frequent to consistent FL-HL coordination (mean score, bicycle, 13.8 ± 1.3, treadmill, 14.0 ± 1.4). Both of the BBB scores in the locomotor trained groups were significantly greater (*p* < 0.05, ANOVA) than the scores determined for the untrained group.

At the final stage of training (week 12), the contused untrained control animals showed frequent to consistent weight supported plantar steps, and no to occasional FL-HL coordination (mean score, 11.25 ± 1.25; Figure 6C). However, in this stage, animals of both trained groups showed consistent FL-HL coordinated and consistent weight supported steps (mean scores, bicycle, 14.25 ± 1.4, treadmill, 15.25 ± 1.7). Moreover, these trained animals showed occasional dorsal stepping and rotated paw positioning during their locomotion. Occasional toe clearance was also observed in some trained animals (in both groups). Please note, the terminologies, never (0%), occasional (less than or equal to half, ≤50%), frequent (more than half but not always, 51–94%), and consistent (nearly always or always, 95–100%) used above, are described in Basso et al. (1995).

In summary, open field locomotor recovery scores scaled at postcontusion weeks 4, 8, and 12 were significantly greater in both of the training groups compared with untrained controls (Figure 6C). The bicycle training group demonstrated the highest recovery score at post-injury 1 month, which was also significantly greater than the treadmill group (Figure 6C). However, at postcontusion 8 and 12 weeks, both training groups showed similar recovery scores (ANOVA).

### Histology and immunohistochemistry

**Figure 7:**
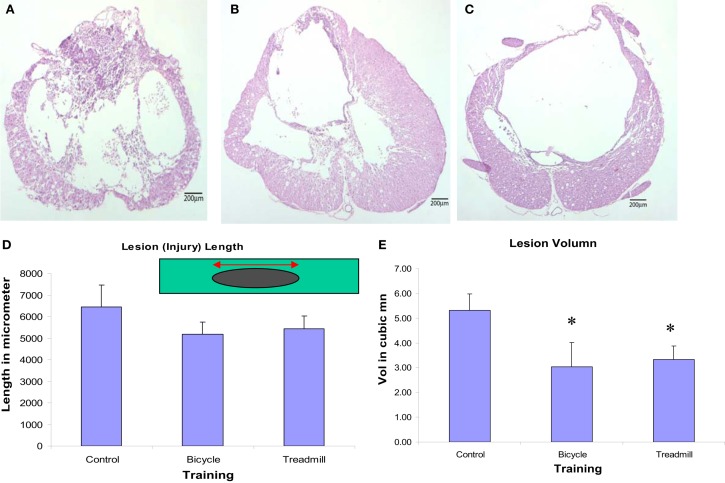
**Light microscopic qualitative studies of spared tissue revealed better preservation of myelin, axons, and collagen morphology in locomotor trained animals (B,C) compared to contused untrained control (A)**. Animals treated with either type of locomotor training revealed decreased lesion length (not significant) **(D)** and significantly decreased lesion volume **(E)** compared with contused untrained control (*p* < 0.05, *n* = 6 in each group; mean ± SEM).

Both locomotor trained groups revealed decreased lesion volumes (rostro-caudal extension) and more spared tissue at the lesion site. Our histological studies indicated that both the injured-bicycle-trained group and the injured-treadmill trained group had shorter lesion lengths, and significant smaller lesion volume than the injured-untrained controls (Figures 7D,E). The measured lengths of lesion for the three different treatment groups showed bicycle-trained rats to have the shortest mean length (5178.3 ± 559.5 μm), followed by treadmill-trained rats (5441.4 ± 549.9 μm), and finally by the control rats (6438.0 ± 1019.1 μm). The difference in lesion lengths among the three treatment groups was not significant, but there was a noticeable trend. The lesion volumes for the three different treatment groups showed bicycle-trained rats to have significantly the shortest mean volume (mean ± SEM; 3.03 ± 0.98 mm^3^; *p* < 0.001 compared to control), followed by treadmill-trained rats (3.33 ± 0.55 mm^3^; *p* < 0.001 compared to control), and finally by the control rats (5.31 ± 0.67 mm^3^). Light microscopic qualitative studies of spared tissue revealed better preservation of myelin, axons, and collagen morphology in locomotor trained animals (Figures 7A–C). Importantly, these data indicate that the therapeutic efficacy of ergonomically practical cycle training was more effective in preserving spared tissue than more labor-intensive treadmill training.

**Figure 8:**
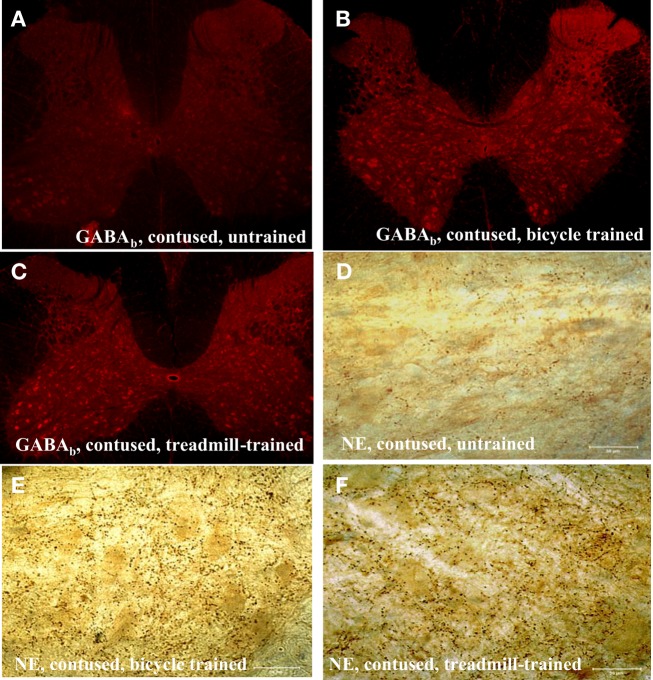
**Immunohistochemistry of lumbar spinal cord showing upregulation of GABA_b_ receptors [5× magnification in (A–C)], and sprouting of dopamine-beta-hydroxylase (DBH) positive noradrenergic (NE) projecting fibers in the ventral horn of the lumbar spinal cord in both types of locomotor training (E,F) compared with contused untrained controls (D; scale bar, 50 μm)**.

**Figure 9:**
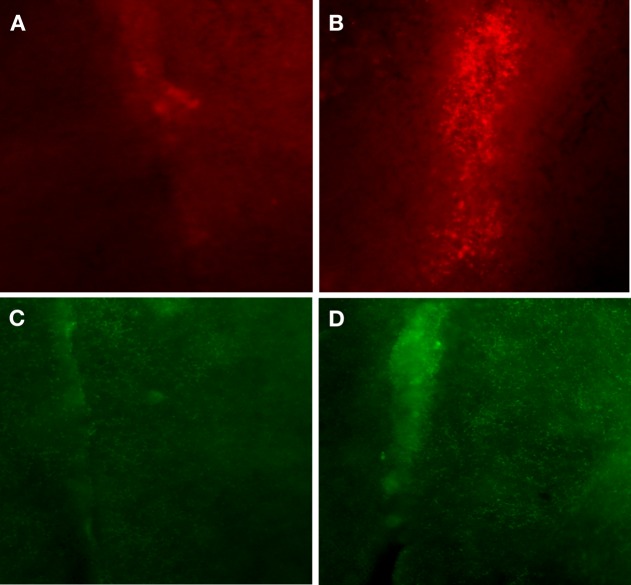
**Immunohistochemistry of thoracic (T_10_) spinal cord (two segments below the injury epicenter), especially areas adjacent to dorsal median septum showed huge upregulation of BDNF (B) following 1 week of bicycle locomotor training compared with contused untrained control (A)**. The GABA synthetic enzyme, glutamic acid decarboxylase (GAD_67_) showed also greater immunoreactivity **(D)** in the same area (1 week bicycle training) compared with untrained contused control **(C**; 20× magnification).

**Figure 10:**
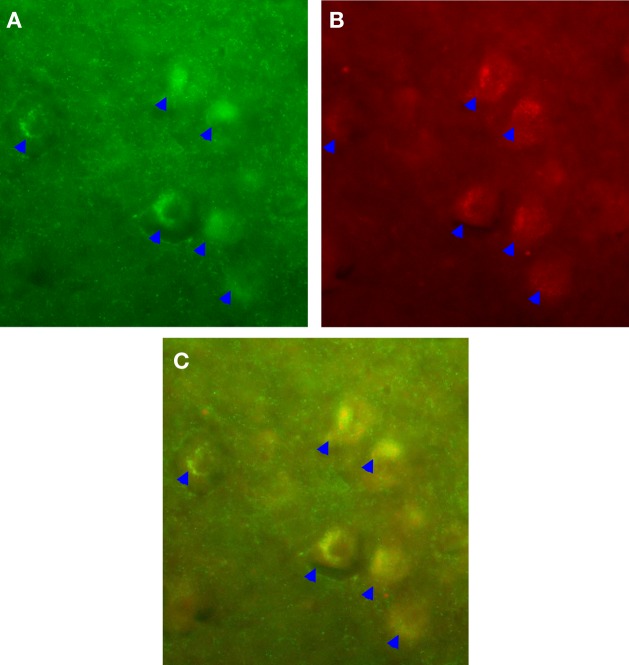
**IHC showed expression and co-localization of GAD_67_ and BDNF in the ventral horn (VH) motoneurons (arrows)**. GAD_67_ showed a diffuse staining in cell bodies and fibers as well as punctate staining in the VH **(A)**. Interestingly, those GAD_67_ immunostained motoneuron cell bodies also stained with BDNF **(B)**; **(C)** is the merged image of **(A,B)** (20× magnification).

We observed a robust increase in the immuno-expression of GABA_b_ receptors and NE fiber sprouting throughout the lumbar spinal gray of both trained animals compared with tissues from untrained animals (Figure 8). Moreover IHC studies indicated upregulation of GAD_67_, and BDNF immunoreactivity at T_10_-T_11_ (immediately below the injury epicenter at T_8_) especially areas adjacent to dorsal median septum and ventral horn (VH) following 1 week of bicycle locomotor training (PO 2 weeks; Figure 9). Interestingly, double IHC showed expression and co-localization of GAD_67_ and BDNF in the VH motoneurons (Figure 10). GAD_67_ showed a diffuse staining in cell bodies and fibers as well as punctate staining in the VH (Figure 10), and those GAD_67_ immunostained motoneuron cell bodies also stained with BDNF (Figure 10C and merged panels of Figures 10A,B).

## Discussion

The present study evaluated the influence of (two types of) locomotor training (treadmill and cycling) on measures of spasticity, reflex excitability, and limb use following injury in a laboratory model of traumatic SCI. These measures included assessment of ankle extensor spasticity; neurophysiological rate-depression of ankle extensor muscle monosynaptic reflexes, lower limb axis, base of support, and open field walking (BBB). Untrained animals with SCI revealed significant locomotor disabilities that were quantitated using these reflex measures. Compared with the untrained injured controls, each of these functional measures was significantly improved in the animals undergoing either of the two types of locomotor training. The three significant findings of these studies: (1) confirmed previous findings regarding significant changes in limb use, spasticity, and reflex excitability of lower limbs following experimental contusion injury of the midthoracic spinal cord, (2) demonstrated significant positive improvement in measures of limb use, spasticity, and reflex excitability by locomotor training, and (3) that the effectiveness of the ergonomically practical cycle training was comparable with treadmill locomotor training in influencing these specific measures.

### Spasticity

In the present study, untrained contused animals revealed a pattern and time course for the development of spasticity that was similar to that we previously reported (Bose et al., 2002). Specifically, at postcontusion week 1, a “tonic” type of spasticity was observed (also in the other two groups, before locomotor training), whereas, at week 2, a suppressed velocity-dependent reflex excitability was detected in untrained contused animals. Finally, by week 4, an enduring, robust, velocity-dependent (dynamic) spasticity appeared. The ankle torques recorded at the lower test velocities (49–272°/s) were not significantly greater than observed in normal controls, nor were these correlated with synchronized EMG activity in the ankle extensor muscles. These low velocity ankle torques were, therefore, interpreted to be contributed by the passive properties of the muscle and joint tissues. By contrast, test rotations at the upper test velocities revealed increased stiffness of ankle rotation that was time-locked to stretch-evoked EMGs recorded from the ankle extensor muscles, indicating resistance contributions from activated ankle extensor muscle stretch reflexes (see also, Bose et al., 2002).

It has been suggested that the alterations in reflex excitability observed following SCI are associated with dynamic changes in the connectivity of the spinal neurons produced by injury-related disruption of descending fibers (Lance, 1981; Young, 1989b). Clinically, these changes in reflex excitability have been reported to include an initial period of hyporeflexia (spinal shock) followed by an enduring hyperreflexia (Kuhn and Mact, 1948; Hiersemenzel et al., 2000). Current evidence suggests that following the initial trauma, many secondary events include membrane damage, systemic and local vascular effects, altered energy metabolism, oxidative stress, inflammation, electrolyte imbalances, unregulated release of neurotransmitters, and a cascade of biochemical changes affect cellular survival, integrity, and excitability (Anderson and Hall, 1993; Tator, 1995; Faden, 1997; see also in, Velardo et al., 2000). Accordingly, alternating patterns of increased and decreased H-reflex excitability have been reported following midthoracic SCI in humans (Diamantopoulos and Olsen, 1967; Hiersemenzel et al., 2000), and in rats (Bose et al., 2002). This initial hyporeflexic state is suggested to be associated the sudden loss of tonic input and/or trophic support from supraspinal to spinal neuronal centers. In humans following midthoracic SCI, the initial period of hyporeflexia has been referred to as “spinal shock.” A recent time course study of H-reflex excitability following human SCI interpreted this period of hyporeflexia as a period of transition that was followed by the appearance of a permanent hyperreflexia (Hiersemenzel et al., 2000). Hutchinson et al. (2001) and others (Gregory et al., 2003; Haddad et al., 2003; Stevens et al., 2006; Liu et al., 2008, 2010; Shah et al., 2008) reported a significant atrophy of the lower limb extensor muscles occurred in the rat during this initial 2 week period following midthoracic contusion SCI. Consistent with these observations, in the present study, the data recorded from the untrained contused animals revealed significant fluctuations in the excitability of lumbar reflexes over a time course of several weeks following midthoracic injury. Interestingly, only 1 week of training (both types) prevented the suppression of ankle extensor muscle stretch reflexes, as observed previously (Bose et al., 2002), and also recorded at postcontusion week 2 in untrained injured animals.

In addition, both treadmill and bicycle training not only prevented the initial hyporeflexic state, but also prolonged the transition to develop a permanent hyperreflexic state (spasticity; Figures 4C,D). At the end of postcontusion week 8–12, significant increases were observed in ankle torque and in the magnitude of the ankle extensor muscle EMGs burst discharge that was time-locked to the ankle rotation. Although, these values were greater compared with pre-injured normal controls, these increased ankle torque and EMGs were significantly lower than that observed in untrained contused controls. Since these increases in ankle torque and EMG were observed only at the higher rotation velocities, it was concluded that these were produced by increased velocity-dependent stretch reflexes of the ankle extensor muscles.

Following chronic SCI, several mechanisms might contribute to the increase in reflex excitability, although the principal common denominators include enhancement of excitatory synaptic input and a reduction of inhibitory control of synaptic input (Katz and Rymer, 1989; Katz, 1999). Excitatory interneurons may become more responsive to muscle or skin afferent activity due to collateral sprouting (Goldberger and Murray, 1988; Krenz and Weaver, 1998), denervation sensitivity (Curtis and Eccles, 1960), and/or changes in presynaptic inhibition (Burke, 1988), or a combination of these changes. In this regard it is noteworthy that our velocity-dependent spasticity measuring instrumentation incorporated both stretch reflex afferent input from the triceps surae muscles as well as input from the skin. The lengthening resistance of the triceps surae muscles was measured by quantitating ankle torque during 12° dorsiflexion rotations of the ankle from 90°. Contact with the foot was achieved using a form-fitted cradle attached with the force transducer and aligned with the dorsal edge of the central footpad 2.6 cm distal to the ankle joint. Therefore, during 12° dorsiflexion of the ankle, afferent input from lengthened muscles and skin afferent input from the footpad were activated. Therefore, the improvement in velocity-dependent ankle torque data represents improvement in spasticitic hyperreflexia elicited by muscle stretch and skin afferent input.

Descending fibers containing NE systems are considered to play important role in the regulation of spinal cord function and in the modulation of spinal reflexes (Fung and Barbeau, 1994; Hasegawa and Ono, 1995; Kobayashi et al., 1996; Li and Zhuo, 2001). Moreover, it has been reported that the synaptic effectiveness of group II afferents is modulated by NE descending neurons (Jankowska and Riddell, 1995). Although, it is not known specifically how locomotor training alters excitatory synaptic input and/or changes in presynaptic inhibition, recent studies have revealed that exercise dependent activity upregulates a host of factors that may contribute to these changes (Gomez-Pinilla et al., 2001; Bose et al., 2005). In addition to presynaptic mechanisms, changes in postsynaptic mechanisms that regulate the input/output gain of motoneuron discharge must also be considered (Kernell, 1979; Hounsgaard et al., 1984; Binder, 2003). Numerous studies have revealed that the gain of synaptic inputs can be amplified up to a factor of five by brainstem/monoaninergic inputs that regulate dendritic persistent inward currents (PICs) utilizing sodium and calcium channels (Schwindt and Crill, 1980; Bennett et al., 1998; Lee and Heckman, 1998a,b, 2000; Perrier and Hounsgaard, 2003; Harvey et al., 2006). The higher the PIC, the higher the synaptic gain and consequent burst rate of the motoneurons. Segmental regulation of PICs occurs through inhibitory mechanisms that regulate afferent inputs (Heckman et al., 2003). It has been proposed that the acute period of hyporeflexia that follows SCI can be attributed to a reduction in dendritic PICs; subsequently, after several weeks, motoneurons re-acquire PICs that can be easily initiated by segmental inputs (Lee et al., 2003). These unregulated PICs are proposed to significantly contribute to clonus and spasms, and associated amplified bi-stable properties of motoneurons. In this context, the segmental inhibitory processes, such as presynaptic inhibition, have an even more important role in the regulation of sensory transmission.

The delayed and milder form of spasticity development that accompanied locomotor training observed in the present studies, could be a result of improved inhibitory mechanisms that regulate afferent inputs. Activity related reorganization of segmental circuitry including descending inputs, segmental synaptic inputs, and local interneurons might contribute in this improvement. Neuronal circuits, stimulated by the proper activation of peripheral afferents via the training, may reorganize by strengthening existing and previously inactive descending connections and local neural circuits. Thus, optimization of neuroplasticity may be a viable foundation for developing rehabilitation strategies that facilitate recovery of locomotion following SCI.

### Reflex excitability

Studies in rats with experimental spinal cord trauma have demonstrated the appearance of progressive changes in processes that regulate transmission in reflex paths to ankle extensor motoneurons (Thompson et al., 1992, 1993, 1998). Particularly evident was a robust decrease in rate-dependent depression tested in reflex pathways to ankle extensor muscles following midthoracic SCI that exhibited a clinical definition of spasticity (Bose et al., 2002). Similar changes have been observed in humans following injury (Ishikawa et al., 1966; Diamantopoulos and Olsen, 1967; Calancie et al., 1993; Schindler-Ivens and Shields, 2000). The loss of rate-dependent depression has been suggested to be associated with injury-induced plasticity of presynaptic processes that regulate afferent transmission to motoneruons (Thompson et al., 1992, 1998; Bose et al., 2002).

There are several important practical reasons for using the plantar H-reflexes (not soleus H-reflexes) in these studies. The distribution of tibial motoneurons innervating the soleus is anatomically continuous with those to the plantar muscles with considerable overlap in the fourth lumbar spinal cord segment (Crockett et al., 1987; Gramsbergen et al., 1996; Homonko and Theriault, 1997). The plantar H-reflexes are far more robust than soleus reflexes in anesthetized animals possible related to the high innervation ratio of plantar muscles (Crockett et al., 1987). Second, with regard to stimulation and recording, the plantar H-reflexes are far more accessible than soleus H-reflexes. Third, the short length of plantar muscles makes compound EMG recordings more robust than compound EMG recordings in the exceptionally long soleus muscle. Therefore, to accommodate weekly, non-invasive recordings (in anesthetized animals), the easily accessed, robust plantar/H-reflexes are far more practical. Further, it is presumed that since parallel changes occurred in both calf and foot muscles following SCI (Thompson et al., 1992), treatment-induced changes may similarly influence both the rostral and caudal portions of the tibial motoneuron pool that innervates these two muscle groups.

This reflex analysis has provided a quantitative assay of changes in inhibitory processes that regulate motoneuron excitability after experimental treatments in animals (Thompson et al., 1993; Skinner et al., 1996) and also in humans following experimental treatments using exercise (Trimble et al., 2001; Kiser et al., 2005) and transplantation (Thompson et al., 2001b). The influence of treatments on rate-sensitive depression has been suggested to be associated with treatment-induced changes in the influence of inhibitory interneurons. Skinner et al. (1996) proposed that continual depression of Ia afferents during cycling exercise in the spinalized rat promoted neural reorganization and preserved the local neural circuitry responsible for presynaptic inhibition, thus normalizing the values of rate-depression observed at rest. A case study by Trimble et al. (1998) provided the first evidence for normalization of rate-sensitive depression following specialized locomotor training in a human after incomplete SCI (Trimble et al., 1998). The data from our studies indicate that the normalization of rate-sensitive depression is associated with an improvement of gait and open field locomotion, and velocity-dependent lengthening resistance of hindlimb extensor muscles. We propose that these task-specific trainings (bicycle and treadmill) provided patterned therapeutic activity in the injured and/or altered neural circuitry and this therapy decreased the maladaptive plasticity that contributes to spasticity and altered locomotor function. Two neurotransmitter systems that appear to play critical roles in the modulation of segmental reflex modulation are GABA and NE. We have observed that the rate-dependent inhibition and velocity-dependent ankle torque are profoundly influenced by GABA_b_-specific agents (Wang et al., 2002; Thompson et al., 2005) and NE-specific lesions (Thompson et al., 1999; Bose et al., 2001). Specifically, l-baclofen (which acts upon GABA_b_ segmental circuitry) increased rate-dependent inhibition and decreased velocity-dependent ankle torque, whereas selective neurotoxic lesions of NE fibers produced non-specific increase in reflex excitability. While the specific role of GABA_b_ receptors is the topic of ongoing research, GABA_b_-mediated synaptic depression using baclofen is currently the most potent and widely used drug for treating spasticity (Penn and Kroin, 1984, 1985; Penn, 1988). It is known that acute baclofen treatment reduces both the monosynaptic and the polysynaptic components of the stretch reflex (Capaday and Stein, 1986; Advokat et al., 1999). We propose that in this study locomotor activity-induced plasticity might up-regulate GABA_b_ receptor and NE mediated inhibition which in turn result in improvement of reflex excitability.

### Limb axis and base of support

Alteration of hind limb axis and base of support following spinal cord contusion have been reported with suggestions that these deficits were produced by injury-related dysfunction of long tracts, as well as injuries of the propriospinal system (Kunkel-Bagden et al., 1992, 1993; Bose et al., 2002). In addition, it has been shown that excitotoxic injury of the L_1_-L_2_ gray matter resulted in locomotor ataxia in rats (Magnuson et al., 1999). That balance deficits can be associated with long tract injury is also suggested by our recent work that reported that specific lesion of the NE descending neurons (using i.t. anti-DBH saporin toxin), resulted in external deviation of the hind limb axis and base of support (Bose et al., 2001). In animals and humans with SCI, previous studies have shown improvements in gait parameters following locomotor training using body weight support on the treadmill and manual assistance, but have not concurrently evaluated effects of bicycle locomotor training following animal SCI. In the present studies, 2–3 months of locomotor training significantly reduced outward deviation of the hind limb and base of support in both treadmill and bicycle training groups compared with untrained contusion group. Interestingly, no significant difference in hind limb rotation or base of support was observed between the two training groups following 2–3 months training. This study so far is the first comparable investigation of two rehabilitation strategies in an animal SCI spasticity model.

### Open field locomotion

Studies progressing over the last two decades have revealed that spontaneous locomotor recovery following SCI is contingent upon the preservation of fibers diffusely located in the ventral caudal – ventro-lateral funiculi of the rat spinal cord (Das, 1987; Brustein and Rossignol, 1999; Basso et al., 2002) or gray matter of the T_13_-L_2_ spinal segments (Magnuson et al., 1999). By contrast, animals with surgical lesions of the dorsal spinal cord at T_8_ that preserved ventral funiculi, demonstrated sufficient self-training that no detectable difference was observed in their locomotor recovery compared with animals that were systematically trained using a treadmill (Fouad et al., 2000). However, following moderate midthoracic contusion injury, injured animals in the present study that received locomotor training revealed greater BBB scores than untrained animals. Longitudinal testing at 4, 8, and 12 weeks following injury revealed that training increased both the rate and magnitude of recovery. It is interesting that recovery scores tested at postcontusion week 4 indicated a particularly robust recovery in the cycle trained group, that was significantly greater than both the untrained and the treadmill trained group. Although the specific reason for this result at this time point is not clear, it is possible that the efficient and uniform pattern inherent to the nature of the cycle training was particularly effective during the early training period. By contrast, animals in the treadmill groups had more freedom to vary limb placement and weight support that could have contributed more variability in the training pattern during this initial period. Moreover, the loading was minimal during the treadmill training, especially in the first 4 weeks. In animals and humans with SCI, previous studies have shown improvements in gait parameters following locomotor training using body weight support on the treadmill and manual assistance (Harkema et al., 1997; Barbeau et al., 1998; Behrman and Harkema, 2000; Dietz and Harkema, 2004; Timoszyk et al., 2005) but have not concurrently evaluated effects of bicycle locomotor training following animal with SCI.

### Histology and IHC

The ventral and lateral WM subserve most of the important hindlimb locomotor function and contain important descending pathways including the rubrospinal tract in the dorsolaeral funiculus and more importantly the reticulospinal tract that is more diffusely distributed in the ventral and lateral WM. Moreover, stride length and base of support have been associated with preservation of the reticulospinal and vestibulospinal pathways for maintenance of posture and trunk stability (Goldberger, 1988).

It is known that transection of the rat spinal cord reduced the binding of [^3^H]GABA by 80% (Chuang, 1989). The decrease in GABA binding below the level of SCI suggests that a decrease occurs in the number of GABA receptors. Most of GABA_b_, a metabotropic receptor, in the spinal cord is presynaptic and located on descending axons, although some of the GABA_b_ receptors are on incoming dorsal root afferent axons (Bowery et al., 1980). Normally, incoming dorsal root information is subject to presynaptic GABA inhibition, which can reduce the amount of excitatory neurotransmitter release (Bowery et al., 1980). Possibly these areas are a source of GABAergic afferents which might participate in the upregulation of BDNF in response to training as seen elsewhere in the CNS following exercise (see review, Cotman and Berchtold, 2002). We suggest that an increase in GAD_67_ leads to increased GABA production in spinal neurons below the injury site, resulting in altered inhibition and trophic support during posttrauma recovery and adaptation. Moreover, locomotor training induced hyperexpression of GAD_67_ might inhibit excitotoxic effects mediated by excitatory neurotransmitters at the site of injury. Increased GABA synthesis around the central canal, in the vicinity of ependymal cells, has been reported as a regenerative process in the mammalian spinal cord (Tillakaratne et al., 2000). This data is important and can be argued that locomotor training induced inhibitory neurotransmitters (GABA/GAD_67_ and NE) and BDNF’s availability could be crucial in reducing the lesion length and volume by optimizing the excitotoxic effects, strengthening neuronal structure, stimulate neurogenesis and increase resistance to further injury. Although exercise mobilizes many gene expression profiles (Cotman and Berchtold, 2002), increased levels of BDNF and inhibitory molecules like GABA and NE could be related to spinal cord plasticity related to post-training improvement of spasticity.

In the present studies, locomotor training-related improvements in spasticity and locomotor recovery were correlated with the decreased lesion volume and more spared white matter. In addition, immunohistochemical studies of these tissues, compared with untrained SCI controls, revealed marked upregulation of BDNF, GABA, and norepinephrine which might account for these decreased lesion volume and more spared tissue.

The findings of the present study are consistent with the suggestions that as therapy, the locomotor training regimen using either treadmill or cycle, promotes the recovery of walking by optimizing the activity-dependent neuroplasticity of the nervous system (Edgerton et al., 1992; Muir and Steeves, 1997; Bose et al., 2005a). Neuronal circuits, stimulated by task-appropriate activation of peripheral and central afferents via the locomotor training, may also reorganize by strengthening existing and previously inactive descending connections and local neural circuits (Edgerton et al., 1992; Dietz et al., 1997; Muir and Steeves, 1997; Barbeau et al., 1998; Basso, 2000). These studies indicate that a locomotor training regimen using either treadmill or cycling significantly enhanced several issues related to locomotor recovery. It is interesting that similar results were obtained with therapeutic locomotor training using either treadmill or bicycle. Although the precise similarities and differences between the two modes of locomotor training have not been systematically quantitated in this animal model, there are some general observations that are relevant. While locomotor exercise is common to both modes of therapy, treadmill walking has an advantage of imposing a higher load, but also, has the disadvantage of a greater variance of locomotor form. On the other hand, cycling offers a lower level of loading, but provides opportunity for greater precision of systematic locomotor form. We propose that this locomotor exercise of either type, benefits from activity-dependent neuroplasticity of the locomotor circuitry, and that the distinct advantages of each mode sufficiently engage the circuitry to induce a positive therapeutic benefit.

The finding presented here is highly significant in terms of translational potential of less labor intensive cycle exercise into clinical use in treatment of human SCI patients in clinic as well as in home setting. This is due to the fact that cycling exercise requires much fewer support personnel and less expensive equipment than does treadmill walking. While treadmill exercise in humans has been shown to decrease the excitability of lower limb reflexes, cycling exercise in humans with both legs has not been tried as much but is promising (Trimble et al., 2001; Kiser et al., 2005). Conceivably, cycling devices for human SCI patients will need to be engineered incorporating appropriate physiological parameters to increase its clinical use.

## Conflict of Interest Statement

The authors declare that the research was conducted in the absence of any commercial or financial relationships that could be construed as a potential conflict of interest.
